# Spaceflight alters reaction time and duration judgment of astronauts

**DOI:** 10.3389/fphys.2023.1141078

**Published:** 2023-03-17

**Authors:** Olga Kuldavletova, Deborah C. Navarro Morales, Gaëlle Quarck, Pierre Denise, Gilles Clément

**Affiliations:** University of Caen Normandy, INSERM, COMETE U1075, CYCERON, CHU of Caen, Caen, France

**Keywords:** duration judgment, internal clock, memory, spaceflight, time perception

## Abstract

We report a study on astronauts aimed at characterizing duration judgment before, during, and after long-duration stays on board the International Space Station. Ten astronauts and a control group of 15 healthy (non-astronaut) participants performed a duration reproduction task and a duration production task using a visual target duration ranging from 2 to 38 s. Participants also performed a reaction time test for assessing attention. Compared to control participants and preflight responses, the astronauts’ reaction time increased during spaceflight. Also, during spaceflight, time intervals were under-produced while counting aloud and under-reproduced when there was a concurrent reading task. We hypothesize that time perception during spaceflight is altered by two mechanisms: (a) an acceleration of the internal clock through the changes in vestibular inputs in microgravity, and (b) difficulties in attention and working memory when a concurrent reading task is present. Prolonged isolation in confined areas, weightlessness, stress related to workload, and high-performance expectations could account for these cognitive impairments.

## Introduction

During long-duration spaceflight, astronauts have reported periods of temporary cognitive impairment, which they often describe as “space fog”, a “sensory saturation”, or a “task distraction at work”. This cognitive impairment is characterized by poor concentration, increased errors, altered time awareness, motor slowing, and difficulty with multi-tasking, which results in diminished ability to perform the tasks for which astronauts have been trained (Kanas and Manzey, 2008). Spaceflight might affect these functions through direct microgravity effects or through stress effects associated with sleep loss, physical fatigue due to workload, over-extended tasking, excessive noise, or the emotional burdens of adapting to the novel, hostile environment.

The control of vehicles and other complex systems places high demands on cognitive and psychomotor functions and could therefore be impaired by the conditions of spaceflight. More specifically, spatial and temporal abilities are particularly important when moving or controlling a vehicle. Previous research suggests that spatial abilities, such as an object’s distance and depth, are underestimated when subjects are in microgravity during parabolic (Clément et al., 2008) and orbital (Clément et al., 2013) flight and in patients with vestibular disorders (Clément et al., 2009). The interpretation of these underestimations is that the adaptive changes in the processing of gravitational information by the neuro-vestibular system alter the construction of spatial maps (Clément et al., 2015; Stahn et al., 2020). In contrast, the temporal abilities have received very little attention during spaceflight. Some previous studies reported underestimates of time durations in weightlessness, suggesting a time-compression effect for spaceflight (Albery and Repperger, 1992; Clément, 2018). When astronauts were asked to perform periodic arms movements with the same rhythm as a metronome and continue after the metronome had been switched off, the variability of inter-response intervals significantly increased during spaceflight (Semjen et al., 1998a; 1998b). During time production tasks, some astronauts overestimated a 2-s interval during a short-duration space flight (Ratino et al., 1988), whereas other astronauts underproduced a 1-min interval, and underestimated intervals in the range of hours during long-duration spaceflight compared to preflight baseline (Navarro Morales et al., 2023).

This study aimed to further evaluate the effects of long-duration spaceflight on time perception. Four prospective temporal tasks were used: two time-interval production tasks (while counting aloud or reading digits), and two time-interval reproduction tasks (reproducing the duration of a visual stimulus while counting aloud or reading digits). Because it is known that attention plays a critical role in time perception (Zakay and Block, 1996), a reaction time task was also used to assess the subject’s attention level during these tests.

## Methods

### Participants

Ten healthy crewmembers (9 male, 1 female; age *M* = 44.1, SD = 4.6) who flew on the International Space Station (ISS) participated in this study. All crewmembers passed a United States Air Force Class III medical examination and had no known history of vestibular or oculomotor abnormalities. 15 healthy subjects (6 females, 9 males; age *M* = 43.2, SD = 18.8) participated in a control study in the laboratory.

The test procedures were approved by the European Space Agency Medical Board and the NASA Johnson Space Center Institutional Review Board and were performed in accordance with the ethical standards laid down in the 1964 Declaration of Helsinki. All subjects provided written informed consent before participating in the study.

### Experimental protocol

Literature data from ground-based studies indicate that time perception may be altered by how the subject counts time (Carlson and Feinberg, 1968), their method of responding (Hornstein and Rotter, 1969), body temperature (Hoagland, 1933), and the time of the day that the test is conducted (Pfaff, 1968). Other variables that have an influence on the perception of time duration include whether the subjects are bored or busy (De Wolfe and Duncan, 1959), the modality (i.e., sound vs. vision) of the stimulus (Goldstone et al., 1959) and possibly the age of the subject (Gilliland and Humphreys, 1943). Most of these constraints were taken into account in our experimental protocol: (a) we used both a production and a reproduction method; (b) there were two attention conditions, i.e., one single-task and one dual-task; (c) we used both auditory and visual instructions; and (d) tests were performed preferably in the morning to avoid fatigue.

The experimental protocol was based on the ground-based studies by Pouthas and Perbal (2004). We used two temporal tasks, i.e., reproduction and production of duration. Subjects performed these tasks in two conditions, a counting condition (single-task) and a concurrent reading condition (dual-task). Nine target durations were used: 2, 3, 5, 7, 10, 14, 20, 28, 38 s. One trial per duration was done during each testing session. The order of the duration to produce/reproduce was randomized.

During the test, subjects wore a head-mounted display (Oculus Rift, Oculus VR, Menlo Park, CA), external noise-cancelling earphones, and used a finger trackball connected to a laptop to report their responses (Navarro Morales et al., 2023). On the ground, this test was performed in the seated upright position; on the ISS, astronauts were in the free-floating conditions. During the free-floating conditions there are no proprioceptive, tactile, or static vestibular cues that participate in spatial orientation. Previous studies have demonstrated that the perception of distance and the depth of objects are altered when free-floating in orbit (Clément et al., 2012; 2013). To investigate the relationship between these changes in spatial perception and changes in time perception, the time perception tests were performed in the same conditions as the previous spatial perception tests.

### Reaction time

Information processing speed was assessed by a simple reaction-time task, in which participants were required to press on the keyboard with the right hand as fast as possible in response to a stimulus (a blue square) that appeared in the center of the computer screen, at a short random inter-stimulus onset interval (ranging from 1,000 to 2,000 ms) or at a long random interval (from 2,000 to 3,000 ms). Thirty trials were done during each testing session and the intervals were all different.

### Duration production

**FIGURE 1:**
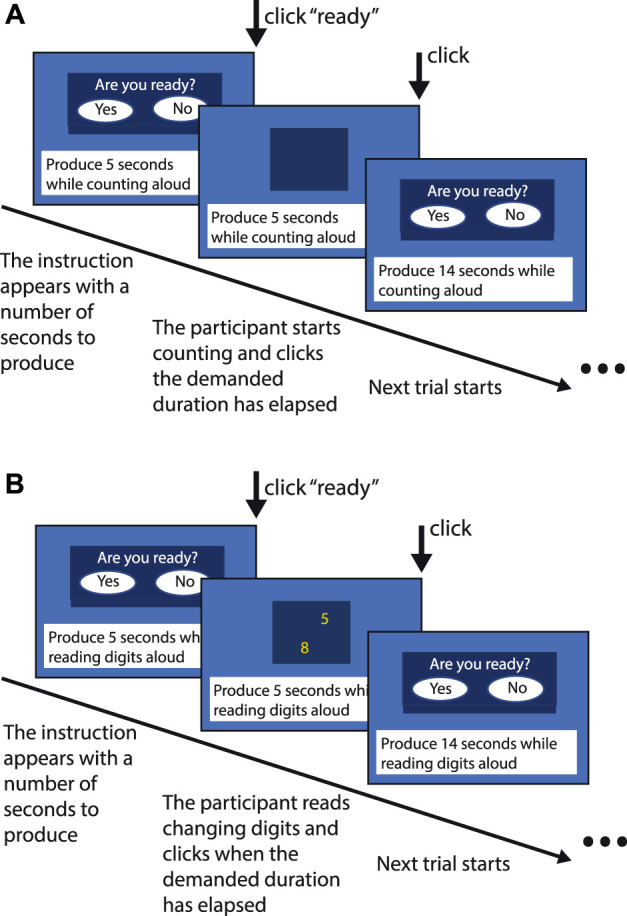
Method used for duration production in the single-task **(A)** and the dual-task **(B)** conditions.

Subjects were instructed to keep the stimulus (blue square) displayed in the center of the computer screen for the target duration given in seconds. At the beginning of each trial, for example the sentence “Produce 14 s” was written at the bottom of the screen and simultaneously pronounced aloud by the computer. Then, the blue square appeared and the subjects were asked to press on the response panel when they judged that the given duration has elapsed (Figure 1).

### Duration reproduction

**FIGURE 2:**
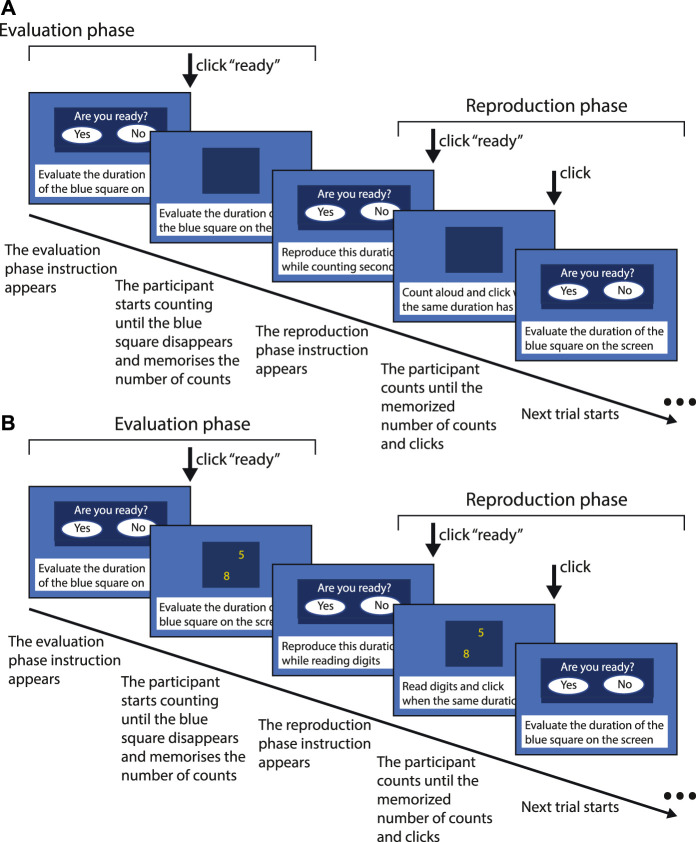
Method used for duration reproduction in the single-task **(A)** and the dual-task **(B)** conditions.

Subjects evaluated the display duration of a blue square presented in the center of a computer screen. At the beginning of each trial, the sentence “Evaluate the target duration” appeared at the bottom of the computer screen and was simultaneously pronounced aloud by a computer-generated voice. Then, the duration was presented (encoding). After the encoding phase, the sentence “Reproduce the duration just evaluated” was displayed and pronounced aloud by the computer. Then, the blue square reappeared and the subjects were asked to press the response panel to erase it when they judged that the previously displayed duration was over (reproduction phase) (Figure 2).

### Single task and dual task

Both duration productions and reproductions were performed using a single and a dual-task. In the single-task conditions, the subjects were asked to count aloud for the stimulus duration as regularly as possible and at the rate they preferred throughout the encoding and the reproduction phases of the reproduction task, and during the production of the stimulus duration, given in seconds, in the production task.

In the dual-task conditions, one-digit numbers were presented in random order in the center of the blue square, with a random inter-digit interval between 350 and 950 ms. Subjects were asked to read aloud these digits.

These two conditions were used to study the effects of attention dedicated to the estimation of duration. The reproduction and production responses in the dual-task conditions require simultaneously evaluating the target duration and performing the concurrent reading task. Therefore, attention is divided between the two types of information processing, which impairs the performance during the tasks. We hypothesized that these impairments would be greater in subjects with reduced attention resources, such as when the astronauts report having difficulties with multi-tasking.

### Study schedule

In the flight study, the tests were performed before, during, and after 6–8-month spaceflights (*M* = 202, SD = 28 days). The preflight test sessions occurred at launch minus (L-) 205 ± 51 days, L-149 ± 55 days, and L-116 ± 45 days. Inflight test sessions were conducted approximately every month: i.e., on flight day (FD) FD17 ± 6, FD46 ± 8, FD71 ± 6, FD99 ± 7, FD134 ± 8, and FD164 ± 7. After the astronauts returned to Earth, tests were performed at return plus (R+) 1 day, R+5 ± 1 day, and R+9 ± 1 day. The order of the tests (reaction time, single/dual duration judgement, single/dual duration production) was randomized consistent across days.

In the control study, the participants performed the same tests as the astronauts using identical hardware and software as on board the ISS. The control subjects were tested during 3 sessions, to compare with the 3 pre-flight sessions with the astronauts. The control subjects' and astronauts' pre-flight sessions were spaced by 44.1 ± 10.2 days and 45.2 ± 28.4 days respectively. The order of the tests (reaction time, single/dual duration judgement, single/dual duration production) was randomized consistent across days.

### Statistical analysis

We examined repeated measures of error in duration production and reproduction (comparing the effects of a single-task and a dual-task) and reaction time before, during, and after spaceflight in 10 crewmembers participating in long-duration missions (>3 months) on board the ISS. Before the flight, crewmembers underwent repeated testing over multiple sessions to allow comparisons with healthy controls and inflight and postflight measurements. The percent errors between the duration judgments and the actual durations were calculated for each target duration, and then averaged for all target durations for obtaining the composite duration percent errors.

Statistical analysis of the data was conducted in R (R Core Team, 2017) using linear mixed-effects models fit by maximum likelihood from the *lme4* package (Bates et al., 2015) to take random factors into account. Analysis of variance was done on the models to assess the fixed factor effects using the Satterthwaite’s method. Pairwise comparisons were made with the *multcomp* package (Hothorn et al., 2008) using the Dunnett method and FDR method *p*-value adjustment.

First, we compared the ground-based responses of the 2 subject groups to establish whether they differed, and whether the results of the 3 test sessions differed. The hypothesis was that the performance of the astronauts on Earth was not different from the control group. An analysis of variance with mixed effects design was used, with reaction time (ms) or duration judgment error (%) as the dependent variable; test sessions (L-205, L-149, L-116) and subject group (astronauts, control subjects) as fixed effects.

For the comparison between pre-, in- and postflight responses in the astronauts, an analysis of variance with mixed effects design was used, with reaction time (ms) or duration judgment error (%) as the dependent variable; test sessions (1 preflight averaged session, 6 inflight sessions, 3 postflight sessions) as fixed effects; subject and target duration as random effects. The Dunnett method was used for pairwise comparison between in- and postflight responses with the preflight responses. The hypothesis was that duration judgment would be different inflight compared to pre-flight and postflight for the time production task, but not for the time reproduction task.

## Results

### Reaction time

**FIGURE 3:**
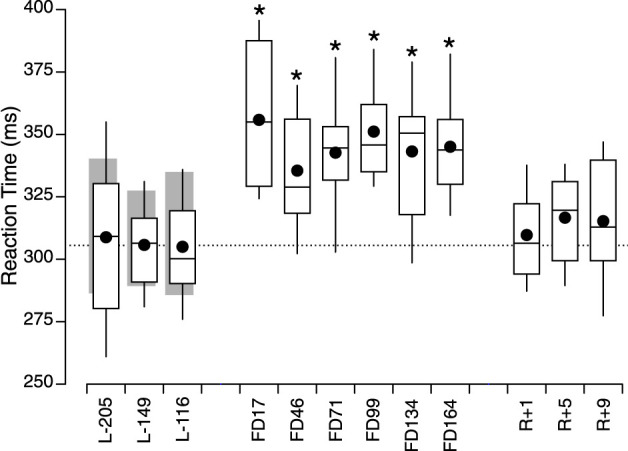
Box and whisker (dash: median, closed symbol: mean, Q1 and Q3) plots of reaction time in 10 astronauts before (L-), during (FD), and after spaceflight (R+). The grey bars show mean ± IQR of the 15 ground-control participants. The dotted line shows the mean of the 3 preflight measures in the astronauts. *p < 0.05 relative to the mean of the 3 preflight measures in the astronauts.

During the three test sessions performed on the ground before the flight, there was no significant difference in reaction time between the astronauts and the control participants [F (25, 1) = 0.242, *p* = 0.626]. There were also no significant differences between the results of the 3 ground-based tests sessions [F (50, 2) = 0.599, *p* = 0.553]. In the astronauts, reaction time was significantly different between sessions [F (90, 9) = 7.241, *p* < 0.001]. The responses during all the inflight test sessions were significantly greater than the mean of the 3 preflight sessions (Figure 3; Table 1).

**TABLE 1:** Post-hoc pairwise comparisons of preflight responses (mean of L-209, L-149, L-116), with inflight and postflight responses in the astronauts (Dunnett test).

Session	Estimate	SD	Z	*p*
**Reaction Time**				
FD17	49.295	9.619	5.125	<0.001
FD46	28.964	9.619	3.011	0.0039
FD71	36.150	9.619	3.758	<0.001
FD99	44.582	9.619	4.635	<0.001
FD134	36.641	9.619	3.809	<0.001
FD 164	38.516	9.619	4.004	<0.001
R+1	3.187	9.619	0.331	0.7403
R+5	10.108	9.619	1.051	0.3771
R+9	8.736	9.619	0.908	0.4092
**Duration Production Single Task**				
FD17	-6.193	1.520	-4.074	<0.001
FD46	-7.711	1.520	-5.072	<0.001
FD71	-4.119	1.520	-2.709	0.0086
FD99	-6.063	1.520	-3.988	<0.001
FD134	-7.870	1.520	-5.177	<0.001
FD164	-8.717	1.520	-5.734	<0.001
R+1	-5.165	1.520	-3.397	0.0010
R+5	-3.826	1.520	-2.517	0.0133
R+9	-2.725	1.520	-1.793	0.0730
**Duration Reproduction Dual Task**				
FD17	-3.940	2.726	-1.446	0.1906
FD46	-4.563	2.726	-1.674	0.1633
FD71	-8.413	2.726	-3.087	0.0087
FD99	-8.111	2.726	-2.976	0.0087
FD134	-8.648	2.726	-3.173	0.0087
FD164	-7.723	2.726	-2.833	0.0103
R+1	-4.370	2.726	-1.603	0.1633
R+5	-2.227	2.726	-0.817	0.4139
R+9	2.232	2.726	0.819	0.4139

### Duration judgment

Preflight, there were no significant differences between astronauts and control participants in duration judgments for the duration production in a single task, [F (25, 1) = 0.013, *p* = 0.910]; the duration production in a dual task [F (24.97, 1) = 0.091, *p* = 0.766]; the duration reproduction in a single task [F (24.97, 1) = 0.001, *p* = 0.976]; or the duration reproduction in a dual task [F (24.74, 1) = 0.0954, *p* = 0.760]. There were also no significant differences between the 3 test sessions on the ground for the duration production in a single task [F (650, 2) = 0.346, *p* = 0.707]; the duration production in a dual task [F (641.82, 2) = 0.304, *p* = 0.304]; the duration reproduction in a single task [F (641.05, 2) = 1.615, *p* = 0.199]; or the duration reproduction in a dual task [F (641.84, 2) = 0.825, *p* = 0.439] (Table 1).

**FIGURE 4:**
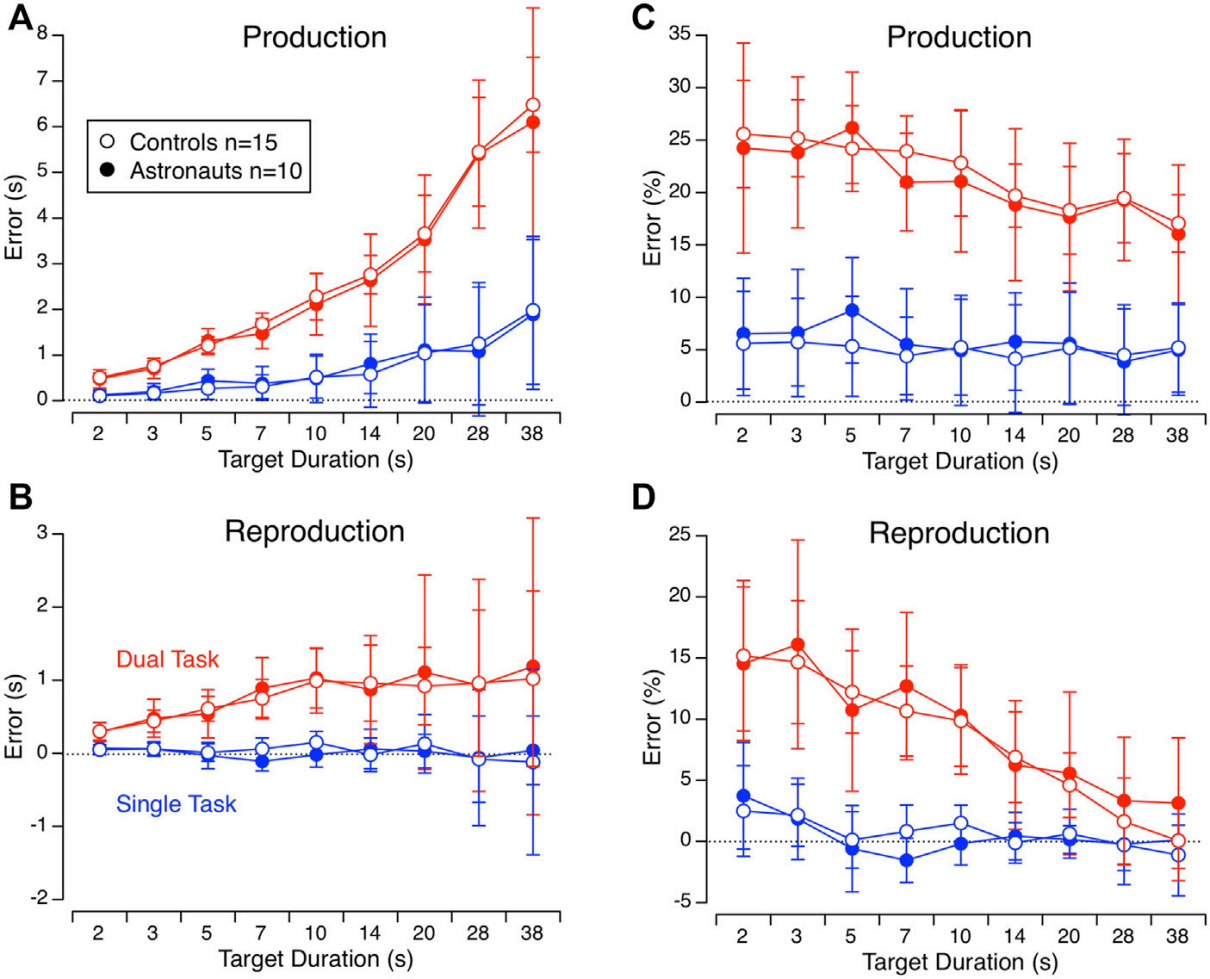
Mean ± SD of error in duration judgments (in s) for the 9 target durations in astronauts and control participants during duration production **(A)** and reproduction **(B)** in the single task (in blue) and the dual task (in red) conditions preflight. **(C**,**D)** Same data expressed in percent error.

During the duration production preflight the subjects overestimated the target duration, and this overestimation was larger in the dual task (20.6 ± 14.3%, mean error ±SD for all target durations) than in the single task (4.7 ± 15.7%) conditions. During the duration reproduction, the subjects were quite accurate in the single-task conditions (0.7 ± 4.4%). However, they also overestimated the durations in the dual-task conditions (9.3 ± 11.7%) (Figure 4).

**FIGURE 5:**
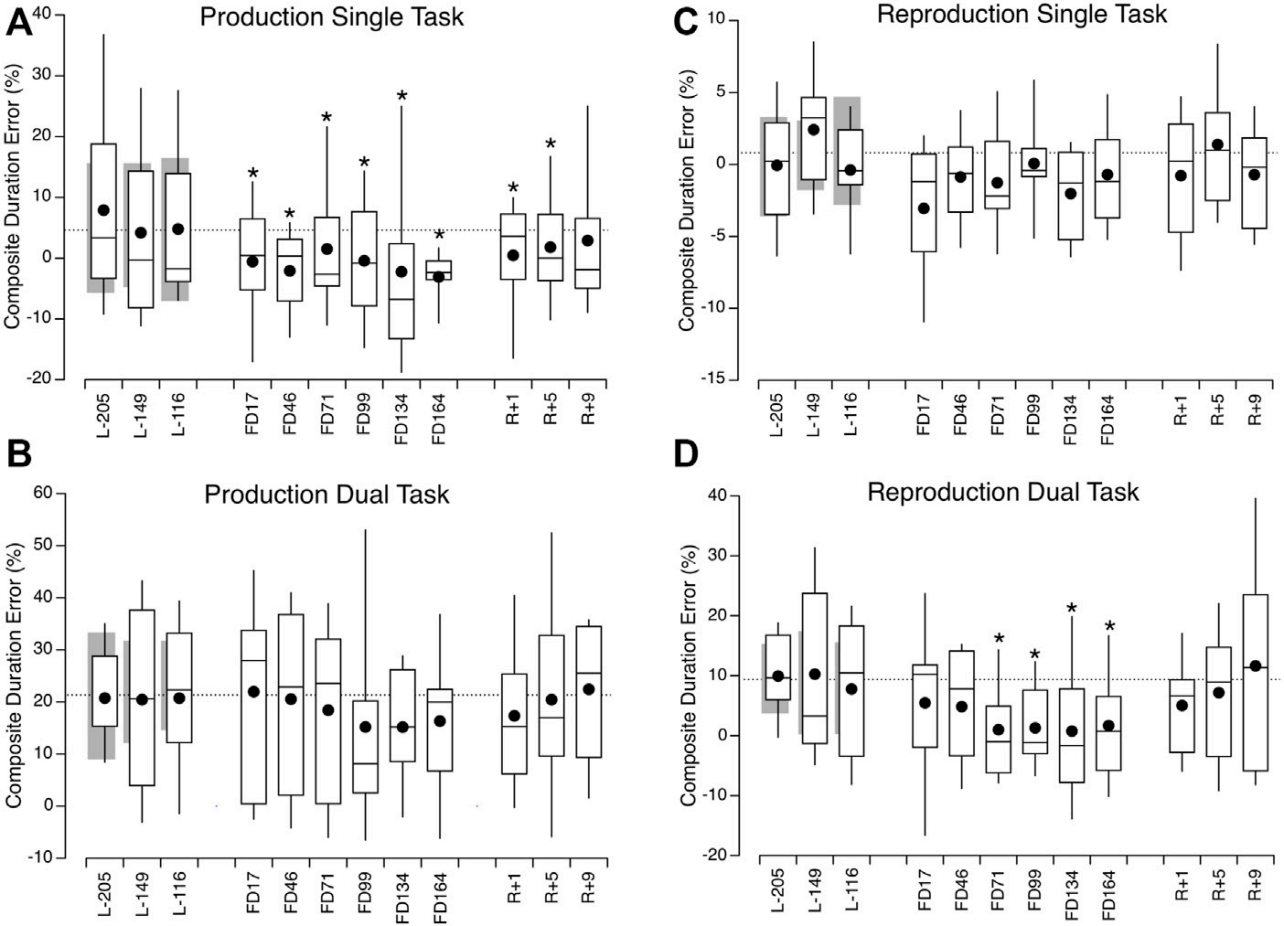
Box and whisker plots of composite duration percent error in 10 astronauts before (L-), during (FD), and after spaceflight (R+) during duration production in the single task **(A)** and dual task **(B)** conditions, and during duration reproduction in the single task **(C)** and dual task **(D)** conditions. The grey bars show mean ± IQR of the 15 ground-control participants. The dotted lines show the mean of the 3 preflight measures in the astronauts. *p < 0.05 relative to the mean of 3 preflight measures in the astronauts.

The duration production single task error (%) differed significantly across sessions [F (890, 9) = 6.129, *p* < 0.001]. The duration judgment errors during all inflight test sessions, as well as during the two first postflight sessions (R+1 and R+5) differed significantly (towards an underestimation) from the mean preflight errors (Table 1). No differences in errors were found between sessions for the duration production dual task [F (881.94, 9) = 1.587, *p* = 0.115] and the duration reproduction single task [F (881.77, 9) = 1.644, *p* = 0.1]. However, during the duration reproduction dual task, the error differed significantly between sessions [F (9, 881.89, 9) = 3.80, *p* < 0.01]. This was particularly the case from FD71 to FD164. The duration judgment error in the postflight sessions was not significantly different from the mean of the preflight measures (Figure 5; Table 1).

## Discussion

This study evaluated reaction time and duration judgments in astronauts during space flight and on the ground, before and after flight. The reaction time increased throughout the flight compared to the preflight level and returned to baseline levels on R+1. When asked to produced durations ranging from 2 to 38 s while counting aloud (single task), the astronauts overestimated these durations before the flight. This overestimation decreased and the astronauts’ judgments were essentially correct during the flight and for a few days after landing. When asked to reproduce a duration while simultaneously reading digits (dual task), the overestimation that was observed on the ground also decreased and the astronauts’ judgments were essentially correct by FD71 and beyond.

### Reaction time

Our results show a 39-ms increase in reaction time during spaceflight compared to pre- and postflight. In contrast, other studies in astronauts have reported no consistent changes in reaction time during a single task (Ratino et al., 1988; Benke et al., 1993; Thornton et al., 1993). This difference could be due to methodological factors in these earlier studies, such as lower number of subjects (from single case to 4) and incomplete data sets (Kelly et al., 2005). Moore et al. (2019) found no change in reaction time postflight compared to preflight, which is in agreement with our results, but they did not measure reaction time during the flight. Other studies have tested astronaut’s reaction time inflight using dual tasks, such as simultaneously aiming and tracking, recognizing numbers, adjusting lines, with conflicting results (Bock et al., 2010; Moore et al., 2019; Takács et al., 2021; Tays et al., 2021; Tu et al., 2022; Weber and Stelzer, 2022). Comparison with the results of these earlier studies is difficult because these tests evaluated additional cognitive resources, such as decision-making and memory, than the reaction time single task in our study. The increase in reaction time during the flight seen in our study can result from numerous factors, among which are diminished attention, or sensorimotor perturbations. “Space fog” is a complex multifactorial phenomenon that could account for the increase in reaction time observed in astronauts during this study. However, space fog seems to be particularly troublesome at the beginning of the flight, with some acute symptoms usually disappearing as the body adjusts to microgravity (Welch et al., 2009). This is differing from our results, which indicate that the increase in reaction time remains relatively consistent throughout the flight.

### Single-task duration judgment

In the duration production single-task condition, the astronauts produced shorter durations inflight compared to the durations produced before the flight. In other words, their subjective time was accelerated. Similar results had previously been observed during short periods of microgravity in parabolic flight (Clément, 2018). Two main models have been proposed to describe on how our brain represents time (Maniadakis and Trahanias, 2014). The first category, also known as extrinsic or centralized models, assumes that the brain uses a time-dedicated neural circuit to encode elapsed time like a clock (Gibbon et al., 1984). In the second category, also known as intrinsic or distributed, time is encoded in the activity of general and inherent property of neurons (Wackermann and Ehm, 2006). Unfortunately, it is difficult to validate these latter models in absence of neuronal activity recordings in humans.

Therefore, we will focus this discussion on how the astronauts’ results could be described using the internal clock model. This model consists of three stages: (a) the first stage includes a pulse generator (or internal pacemaker) whose activity is modulated by attention and arousal, and an accumulator which counts the number of pulses; (b) the second stage is where the pulses reach the working memory module; and (c) the third stage is where decision mechanisms compare the pulses accounted with previous lived events. An increase in the pace of the pulse generator, or an increase in the efficiency of the accumulator, will lead to the perception that external events are slow and to the production of shorter-than-demanded time intervals (Church, 1984).

Performance in the duration production task depends on the speed at which pulses are accumulated, in other words on clock speed and information processing speed. In addition, this task not only requires short-term (working) memory storage in order to maintain the temporal information (i.e., time basis pulses) throughout the trial, but also necessitates long-term memory in which the representation of several durations will be stored. Because the durations to produce are given in conventional units of time (seconds), this long-term memory can be viewed as semantic memory (Pouthas and Perbal, 2004). The underestimation in the duration production in astronauts could thus be interpreted by such an increase in the frequency of the pulse generator. The results of another space study also support a potential acceleration of the internal clock in microgravity. In the Semjen’s (1998a), Semjen’s (1998b) study mentioned earlier, astronauts tapped with a higher frequency when the metronome was turned off while in space compared with their pace on the ground.

Performance in the duration reproduction task not only requires short-term memory storage during the encoding of the target duration, but also necessitated retrieval from long-term storage, because of the limited capacity of short-term memory (Pouthas and Perbal, 2004). Our results did not indicate any change in the duration reproduction single task throughout the flight. This finding is also compatible with the internal clock model, which predicts that duration reproduction tasks are insensitive to the pacemaker-accumulator rhythm alterations (Church, 1984). Indeed, an increase of the rate of the internal clock pulses would lead to an overestimation of the target duration during the evaluation phase, as more pulses would accumulate, which would then be compensated by a “faster” production in the reproduction phase. In other words, what is reproduced by the subjects are not seconds or other arbitrary units, but the number of internal clock pulses, which will match in the evaluation and reproduction phases regardless of the rate of the internal clock.

### Dual-task duration judgment

Assuming that the pacemaker rate was accelerated by spaceflight, then the duration production should be underestimated during spaceflight in the dual task as in the single task conditions. Such change was not observed in our study. Nevertheless, the overall errors in the dual task are much larger than the errors in the single task, and the variability across subjects is also larger in the dual task than in the single task. It is possible that other mechanisms than the acceleration of the internal clock could also affect the response. “The attentional gate model” proposes that the greater attention on time, more pulses are counted and time seems to slow down. Conversely, with distractions, fewer pulses are counted (or pulses are missed), and time seems to be accelerated (Zakay and Block, 1996). The increase in reaction time during the flight also testifies to a decrease in attention. Dual-tasking is cognitively and attentionally challenging. Attention difficulties are commonly been reported during spaceflight (Welch et al., 2009), which makes dual-tasking even more challenging. Different levels of attention in our subjects could account for greater variability in responses, which would make it more difficult to observe significant changes in our measures.

The duration reproduction dual task was the most cognitively demanding of the 4 tasks used in this study. Participant had to (a) estimate how much time had elapsed; (b) retrieve the time stored on the working memory and compare it to the time elapsed; and (c) read digits. Given that microgravity impairs attention (as indicated by the increased reaction time inflight), the cognitive demand increases even more. Nevertheless, we observed smaller errors in duration reproduction in the dual task in the astronauts from FD71 through the remaining inflight sessions compared to preflight. In fact, the duration judgments tended to be underestimated compared to preflight. Ground-based studies have shown that time intervals tended to be underestimated when the difficulty of the dual task increased (Brown, 1997; Block et al., 2010). Other ground-based studies have found that the ability to reproduce a previously experienced duration is largely affected by attention and working memory abilities (Baudouin et al., 2006; Broadway and Engle, 2011). Pouthas and Perbal (2004) have observed that amnesic patients and elderly participants under-reproduce time intervals in dual-tasks, but their time productions don’t differ from control subjects. Since the duration production in a dual task does not require working memory abilities, these authors conclude that this underestimation of duration is due to an inadequate retrieval of information in the episodic memory. A strong correlation between the accuracy of the duration estimates and the responses to neuropsychological memory test in these patients reinforces this interpretation (Pouthas and Perbal, 2004).

The results of 29 studies performed on 32 crewmembers during short-duration flights indicate some cognitive performance degradation in the space environment. Choice reaction time, memory, reasoning, attention switching, pattern recognition, movement time, and dual task performance, all indicated some impairment (Casler and Cook, 1999). Astronaut’s core body temperature increase during spaceflight, which could impair physical and cognitive performance (Stahn et al., 2017). Space motion sickness may have been a contributor to impairments observed in choice reaction time and memory, but other impairments were observed after space motion sickness symptoms had vanished. The deficits tend to resolve in 3 weeks. However, it is not known whether impairment persists during long-duration missions since most of the above studies were performed during flights ranging from a few days to a few weeks only. The lack of long-term studies is an issue because any effects on cognitive abilities should be more intense during longer stays (Manzey and Lorenz, 1998; Fowler et al., 2000). Tests performed with one cosmonaut during a 438-day spaceflight using a dual task with a simultaneous memory search indicated that there were significant deficits during the first month of the spaceflight. Also included in this study were measures to assess the subjective emotional balance and fatigue of the participant. An analysis showed that these measures were correlated with dual-task performance (Manzey et al., 1998).

### Time and space in the brain

Brain imaging studies in returning astronauts have shown that spaceflight disrupts the connectivity in the right temporo-parietal junction (TPJ) (Van Ombergen et al., 2017). This brain area has been shown to be involved in time interval judgments and timing (Bosco et al., 2008; Davis et al., 2009). Lesion of the right TPJ or transcranial direct current stimulation of the right TPJ in healthy subjects were found to impair their spatial and temporal perception (Kaski et al., 2016; Dalong et al., 2021). Patients with right temporal resection lesions also present an acceleration in their internal clock during time production tasks (Pouthas and Perbal, 2004). It has been suggested that the disruption of the connectivity in the right TPJ in microgravity is related to changes in vestibular function (Van Ombergen et al., 2017). Indeed, the TPJ participates in the integration of multisensory modalities and in gravity estimation, and it receives constant stimulation from the vestibular receptors. In patients with unilateral vestibular hypofunction, vestibular perception may be persistently impaired in the duration domain, even when the other domains, such as position and velocity/acceleration perception, remain intact (Kwon et al., 2022). Vestibular stimulation has been found to affect the time perception in healthy subjects (Utegaliyev et al., 2022). Weightlessness unloads the graviceptive part of the vestibular system, significantly altering the sensory inflow to the brain areas treating the vestibular information, including the TPJ. Therefore, these alterations in the sensory flow might affect the function of this area, altering spatial judgments, such as the perception of distance, object size, and motion (Clément et al., 2016; Clément et al., 2015; Clément et al., 2013; Clément and Wood, 2014) as well as temporal judgments (Clément, 2018).

It has even been proposed that time perception is a way for the brain to evaluate the aspects of Newtonian dynamics and is therefore contributing to its internal models to estimate gravity (Lacquaniti et al., 2015). A recent study by Gravano et al. (2021) showed that astronauts estimated the duration of an imaginary ball motion differently inflight than before flight. The estimated duration of imaginary ball motion represents an equivalent of duration judgment but based on an internal representation of object dynamics rather than on external inputs, as is the case in the present study. However, the authors did not find a statistical difference between the perceived ball motion duration inflight and on the ground, suggesting that adaptation to spaceflight did not affect the internal representation of elapsed time, but affected the astronaut’s movements.

In conclusion, this study shows an alteration of the time perception during spaceflight, which could be due to multiple mechanisms such as the acceleration of the internal clock and degradation of attention and memory. These changes might be provoked by the stress due to isolation in confined areas, heavy workload, and high-performance expectations, but also by the modifications of the vestibular inputs in weightlessness. Future studies on reaction time and duration judgement would benefit from collecting subjective reports or objectives measures of what astronauts refer as space fog to compare the results on a temporal scale.

## Data Availability

The raw data supporting the conclusions of this article will be made available by the authors, without undue reservation.
